# Supplementary material for: Multiplex Assay for Rapid Detection and Analysis of Nucleic Acid Using Barcode Receptor Encoded Particle (BREP)
Source: Biomedicines. 2022 Dec 13;10(12):3246. doi: 10.3390/biomedicines10123246 (PMC9775236; doi:10.3390/biomedicines10123246)
Supplement: Supplementary file 1 [file biomedicines-10-03246-s001.zip › biomedicines-2034585-supplementary.pdf]

biomedicines

Supplementary Information

Multiplex assay for rapid detection and analysis of nucleic acid using Barcode Receptor Encoding Particle (BREP)

Semyung Jung 1, Ki Wan Bong 1*, and Wonhwi Na 2*

1 Department of Chemical and Biological Engineering, Korea University, Seoul 02841, Republic of Korea
2 Engineering Research Center for Biofluid Biopsy, Seoul 02841, Republic of Korea
* Correspondence: bong98@korea.ac.kr (K.W.B.); whna@korea.ac.kr (W.N.)

Table S1. List of primer, Barcode probe, Barcode receptor, and target DNA template sequences used in this study.
Figure S1. Validation of amplification step with agarose gel electrophoresis (LOD).
Figure S2. Validation of amplification with agarose gel electrophoresis (Clinical samples from malaria patients).

Table S1. List of primer, Barcode probe, Barcode receptor, and target DNA template sequences used in this study. (R: Reverse primer; F: Forward primer; B.P: Barcode probe; B.R: Barcode receptor; Target: Target DNA template) Barcode receptor include Quencher (BHQ-1) at 5′ end, biotin at 3′ end, and Reporter (FAM) between the 5'-end and the 3'-end. The underlined character indicates the Barcode region of the Barcode probe and the C-barcode region complementary to the barcode region. Bold indicates the restriction region.

P.f	R	5′-GTCATAAGTGTTTGAACCACTTAC-3′
	F	5′-GGATCCATGGCACCAAAAGCAAAAAT-3′
	B.P	5′-GAATAGTGCGATTCATGCCACATGGAAAAGCTTTAGA[C3spacer]-3′
	B.R	5'-[BHQ-1]<br>AGGACG**GGATCC**ACA[FAM(dT)]TGAATCGCACTATTCTTTTTTTTT[biotin]-3'
	Target	5′-<br>ATGGCACCAAAAGCAAAAATCGTTTTAGTTGGCTCAGGTATGATTGGAGGAGTAA<br>TGGCTACCTTAATTGTTCAGAAAAATTTAGGAGATGTAGTTTTGTTC<br>GATATTGTAAAGAACATGCCACATGGAAAAGCTTTAGATACATCTCATACTAATGT<br>TATGGCATATTCAAATTGCAAAGTAAGTGGTTCAAACACTTATGAC -3
P.v	R	5′-TCGCCCTCTACTGCAGCATC-3′
	F	5′-TGGGACTGTAACACTAAGAAGG-3′
	B.P	5′-GGAACCGGAAGAAGAGTATACCAGATCGAAGATATCAATTATG[C3spacer]-3′
	B.R	5'-[BHQ-1]<br>AGGACG**GGATCC**ACACCG[FAM(dT)]TCTTCTTCCGGTTCCTTTTTTTTT[biotin]-3'
	Target	5'-<br>TCGCCCTCTACTGCAGCATCATAAATAAGTTTCCTTTTCAAATATAATTTTCGAAAT<br>GTTATATCACTATGAAAATTTGTGTCTGTATTATTTACCAAATTC<br>GTAAGTTCCTTCATACATAATTGATATCTTCGATCTGGTATACAAACATCCTTCTTA<br>GTGTTACAGTCCCA-3'
P.o	R	5′-CCTTTACCCTTGTCCCCACT-3'
	F	5′-GGCAGTGATAGCATTGACGA-3
	B.P	5'-CTATCCTTGTTGCTT GCATGGCGTTGGTGTGCATACCAG[C3spacer]-3'
	B.R	5'-[BHQ-1]<br>AGGACG**GGATCC**ACACCG[FAM(dT)]AAGCAACAAGGATAGTTTTTTTTT[biotin]-3'

	Target	5′-<br>GTTAAGAACACTAGTGACAGTGAGAGCATCGACGATACGAATAGCTCTAGTGAGA<br>ATATCAGCAATGTGAATAGTGCTTGTTCGCATGGCGTTGGTGTGCATACCAGTGAT<br>GAAGAAACCACTGTTACGGATTACAGTGATGAAGAAACCGCTGTTACGGGTTCCA<br>TTGATGA<br>AGAAACCACTGTTACGGATTACAATGA-3′
P.m	R	5′-GCCCTTATACCACTTCCACAA-3'
	F	5′-GCAGCAGGAAATGAAAAAGC-3'
	B.P	5'-TCTGGTATCGTGTTC AGTATTACGGAGGAATGGTCACCA [C3spacer]-3'
	B.R	5'-[BHQ-1]<br>AGGACG**GGATCC**ACACCG[FAM(dT)]GAACACGATACCAGATTTTTTTTT[biotin]-3'
	Target	5′-<br>CAGGAAGAAAATAATGATTCGTCTAATGGTCCATCTGAAGAACATATAAAGAATT<br>ATTTAGAAAGTATTCGTAATAGTATTACGGAGGAATGGTCACCATGTAGTGTAACT<br>TGTGGAAGTGGTATAAGGGCTAGAAGAAAGGTTGATGCAAAAAATAAGAAACCT<br>GCAGAATTAGTTTTAAGTGACCTTGAAACTGAAAT-3′

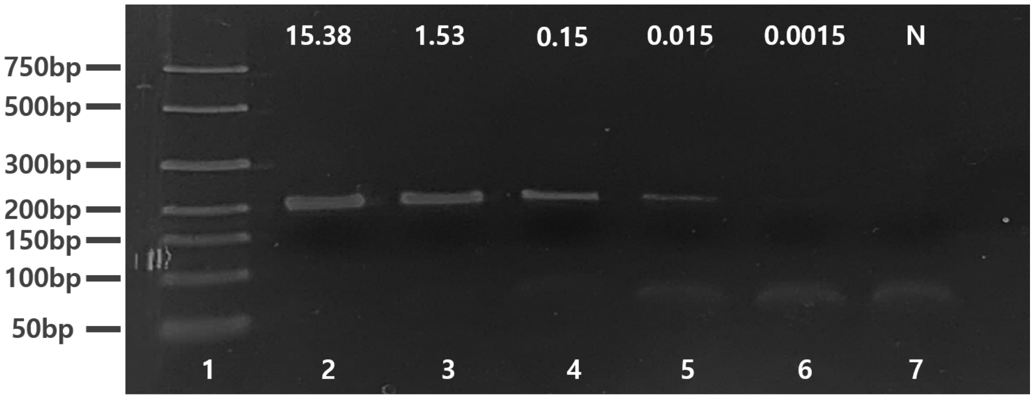

Figure S1. Validation of amplification step with agarose gel electrophoresis (LOD). Lane 1 was 50bp DNA ladder; lane 2-6: amplification step product with 15.38, 1.53, 0.15, 0.015, and 0.0015 pM of DNA template; lane 7 was negative control with deionized water.

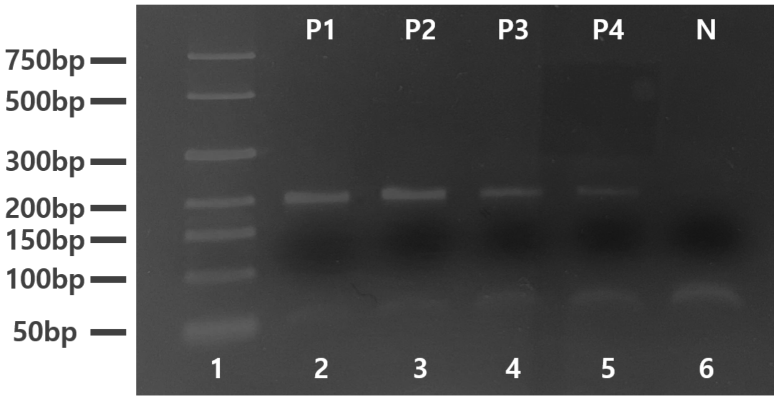

Figure S2. Validation of amplification with agarose gel electrophoresis (Clinical samples from malaria patients). Lane 1 was 50bp DNA ladder; lane 2-5: amplification step product with clinical sample from different patients; lane 6 was negative control with deionized water.
